# 1146. *Lactococcus* species Catheter-Related Bloodstream Infections in Pediatrics: A Case Series

**DOI:** 10.1093/ofid/ofab466.1339

**Published:** 2021-12-04

**Authors:** Sarah E Firmani, Holly Maples, Archana Balamohan

**Affiliations:** 1 Arkansas Children’s Hospital/UAMS, Little Rock, Arkansas; 2 University of Arkansas For Medical Sciences, College of Pharmacy, Little Rock, Arkansas; 3 University of Arkansas for Medical Sciences/Arkansas Children’s Hospital, Little Rock, Arkansas

## Abstract

**Background:**

Central venous catheters (CVC), may lead to central line-associated blood stream infections (CLABSIs). In the past, *Lactococcus* species have seldom been considered pathogenic. However, clinically significant infections have been reported, of which few are pediatric cases, all outside the United States.

**Methods:**

We retrospectively identified pediatric patients with bacteremia secondary to *Lactococcus* spp. admitted to a tertiary pediatric hospital from January 2018 - December 2020. We reviewed the PubMed database for cases of pediatric *Lactococcus* spp. infections in English, peer-reviewed literature.

**Results:**

We identified 3 patients with *Lactococcus spp.* bacteremia. The average patient was 17 months old (range, 6–24 months). All had a CVC; two had short bowel syndrome and 1 had nephrotic syndrome. None received probiotics. Empiric treatment for all included vancomycin. Two of 3 patients were de-escalated to ceftriaxone. All isolates were susceptible to penicillin. Duration of treatment was 10-14 days. Two of 3 were managed with CVC retention and none had recurrence of infection.

A literature review revealed 9 additional cases (Table 1). The most common source of infection was blood (33%), with 66% (2/3) occurring in patients with central lines. Other sources included liver abscess (11%), brain abscess (11%), cerebrospinal fluid (11%), urine (11%), and endocarditis (22%). Median patient age was 12 months (range, 14 days-14 years). Five of 9 patients had an underlying risk factor. Duration of therapy ranged from 7-40 days. Most definitive treatment regimens consisted of a third-generation cephalosporin (44%). Of bacteremia, 2/3 received vancomycin as part of their definitive therapy. Five of 9 reported quantitative antimicrobial sensitivity testing (AST) or interpretation of AST to beta-lactam antibiotics; 80% (4/5) were susceptible.

**Conclusion:**

To the best of our knowledge, these are the first reported pediatric cases of *Lactococcus* infections in the United States and suggests *Lactococcus spp.* should be considered pathogenic in the appropriate circumstances. This series adds to the limited literature, including AST. Continued accrual of susceptibility data may raise the possibility of using a 3^rd^ generation cephalosporin as empiric therapy for Lactococcus bacteremia.

**Disclosures:**

**All Authors**: No reported disclosures

